# Association between Frequency of Breakfast Consumption and Insulin Resistance Using Triglyceride-Glucose Index: A Cross-Sectional Study of the Korea National Health and Nutrition Examination Survey (2016–2018)

**DOI:** 10.3390/ijerph17093322

**Published:** 2020-05-10

**Authors:** Hye Jin Joo, Gyu Ri Kim, Eun-Cheol Park, Sung-In Jang

**Affiliations:** 1 Department of Public Health, Graduate School, Yonsei University, Seoul 03722, Korea; hjjoo22@yuhs.ac; 2 Institute of Health Services Research, Yonsei University, Seoul 03722, Korea; GYURIKIM@yuhs.ac (G.R.K.); ecpark@yuhs.ac (E.-C.P.); 3 Department of Preventive Medicine, Yonsei University College of Medicine, Seoul 03722, Korea

**Keywords:** insulin resistance, triglyceride-glucose index, type 2 diabetes mellitus, breakfast, meals, public health, Korea

## Abstract

Diabetes mellitus is an important chronic disease causing economic and social burden. Insulin resistance is a determinant of diabetes, and regular eating patterns are an important factor in blood sugar control. This study investigated the association between breakfast frequency and the risk of increased insulin resistance in Koreans. Data for 12,856 participants without diabetes in the 2016–2018 Korea National Health and Nutrition Examination Survey were analyzed. Insulin resistance was assessed using the triglyceride-glucose (TyG) index, while the median TyG index value was used to define higher (≥8.5) vs. lower (<8.5) insulin resistance. Association between breakfast frequency and risk of increased insulin resistance was investigated using multiple logistic regression. Compared with those who had regular breakfast 5–7 times per week, the odds ratios (95% confidence intervals) of individuals who did not eat breakfast were the highest at 1.42 (95% CI = 1.24–1.64, *p* ≤ 0.0001). Those who had breakfast 1–4 times per week had an odds ratio of 1.17 (95% CI = 1.03–1.32, *p* = 0.0153). We found that a lower weekly breakfast consumption was associated with a higher risk of insulin resistance in Koreans. Promoting the benefits of breakfast can be an important message to improve the health of the population.

## 1. Introduction

The incidence and prevalence of type 2 diabetes mellitus (T2DM) continue to increase worldwide [1,2]. Diabetes is a chronic disease requiring long-term management and has a high risk of complications [3]. However, in the prediabetes stage, lifestyle changes and weight loss can often reverse the blood glucose level to normal. Hence, early management in the prediabetes stage is important [4]. Therefore, estimating the risk of diabetes in the general population is an important step in minimizing the high morbidity and socioeconomic costs associated with this disease. T2DM is characterized by four major metabolic abnormalities: obesity, impaired insulin action, insulin secretory dysfunction, and increased endogenous glucose output [5,6]. The onset of diabetes is closely related to insulin resistance [7].

Insulin resistance refers to the condition in which the body’s response to insulin is insufficient; the body does not detect an increase in insulin secretion, and the insulin activity may also be ineffective. When the body loses the ability to control the glycemic index, the body’s cell and metabolic functions cannot effectively reach glucose homeostasis. When insulin resistance is high, the body continues to produce insulin even though there is a sufficient level of glucose. As a result, the beta cells that produce insulin in the pancreas are overworked and eventually fail to function. Thus, insulin resistance is a fundamental aspect of the etiology of T2DM and is also linked to a wide array of other pathophysiologic sequelae, including hypertension, hyperlipidemia, atherosclerosis, and polycystic ovarian disease [8].

The gold standard test to assess insulin resistance is the euglycemic–hyperinsulinemic clamp, which measures peripheral glucose uptake under conditions of elevated insulin concentrations [9,10]. However, because the glucose clamp is time-consuming, costly, and complex, it is difficult to apply it in large population studies and in clinical settings. There are simple, reliable, and reproducible indices to measure insulin resistance, such as the homeostasis model assessment—insulin resistance (HOMA-IR) index [11], the McAuley index, and the quantitative insulin sensitivity check index (QUICKI). Nevertheless, several previous studies have evaluated the use of triglyceride-glucose (TyG) indicators as an easy-to-use and accurate predictive method [12,13]. As insulin testing is expensive and is not available in most developing countries, the TyG index is a simple blood test that calculates blood glucose and triglyceride levels, and is a useful indicator for diabetic patients [14]. This makes it an easy and accurate method for screening those who are at a higher risk of developing diabetes, and it can be used in population surveys. In addition, studies using the TyG index in the Korean population showed that the prevalence of coronary artery calcification or arterial stiffness increased significantly as the TyG index increased [15,16], and the index was identified as a useful tool for evaluating insulin resistance [12,17].

The lifestyles associated with diabetes include irregular eating habits or sleeping patterns, smoking, alcohol consumption, and lack of exercise. In modern society, eating habits have become of increased interest and many people are focused on high-quality meals and numerous dietary patterns [18]. However, the importance of mealtime frequency is often overlooked. Some evidence suggests that breakfast may have a greater impact on health than lunch or dinner, because skipping breakfast can reduce the 24 h energy expenditure and elevate the level of blood glucose [19,20]. Additionally, many studies have investigated the relationship between skipping breakfast and health status [21,22,23,24,25,26,27,28]. Several studies have shown that skipping breakfast is associated with depression [21,29], obesity, diabetes, high blood pressure, metabolic diseases [22,23], and CVD [24]. Breakfast can help people obtain a variety of nutrients, resolve energy imbalances, and help with weight loss and weight management [30]. It is also associated with a reduced risk of developing a variety of diseases, such as diabetes, dyslipidemia [31], and cardiovascular disease (CVD) [24,32]. Therefore, having breakfast regularly is an important determinant of health promotion. As a result, health policies to reduce the rate of those who skip breakfast have been implemented in many countries [33]. The findings that skipping breakfast is associated with an increased risk of T2DM in both men and women are in agreement across studies [34,35,36]. Furthermore, skipping breakfast has been suspected to be a risk factor for T2DM, but the associations are not entirely consistent across ethnicities or sexes, and the issue has not been adequately addressed in the Korean population.

We hypothesized that those who did not regularly eat breakfast would have increased insulin resistance and determined that the most useful indicator of this resistance would be the TyG index, which allows for measurement in large population-based studies. Therefore, the purpose of this study was to investigate the link between the frequency of weekly breakfast consumption and insulin resistance calculated by the TyG index in the Korean population.

## 2. Materials and Methods

### 2.1. Data Collection and Study Population

Data were derived from the Korea National Health and Nutrition Examination Survey (KNHANES, 2016–2018) [37]. KNHANES is a nationally representative, cross-sectional study designed to assess the health and nutritional status of the non-institutionalized Korean population aged 1 year and above. KNHANES employs a stratified multistage cluster sampling design based on geographic area, sex, and age to select household units. The initial study population comprised 24,269 individuals. We excluded patients who were diagnosed with diabetes from a physician or had used medication for diabetes and had HbA1c levels greater than 6.5% (48 mmol/mol) or blood glucose values greater than 126.0 mg/dL, in order to conduct a study on people without diabetes. The subjects with missing data for every meal frequency for one week in the last year; missing levels of triglyceride, glucose, and HbA1c; and missing age, sex, marriage status, household income, occupation, education level, region, smoking behavior, alcoholic behavior, physical activity, body mass index (BMI), waist circumference, and subjective health status information were also excluded from the study. Finally, data on 12,856 participants were included in the study.

The KNHANES is a nationwide survey conducted by the Korea Centers for Disease Control and Prevention (KCDC) and in accordance with Article 16 of the National Health Promotion Act, which is a reliable statistic used to assess the health and nutritional status of Koreans. KNHANES data are a valuable resource used to establish health policies tailored to the health status of Koreans. KNHANES data are publicly available and ethical approval is not required to use the data for research. In addition, since the respondents’ information was kept completely anonymous, this study did not require informed consent from the respondents.

### 2.2. Insulin Resistance Using the TyG Index

The main objective of this study was an assessment of insulin resistance using the TyG index, which is a product of the fasting levels of triglycerides and fasting glucose, which is a useful indicator for assessing insulin resistance [38]. In the KNHANES data, fasting blood (fasting after 7 pm on the day before the investigation) was used for tests. The TyG index was calculated as the ln(triglycerides (mg/dL) × fasting blood glucose (mg/dL)/2) [14]. The TyG index is expressed by a logarithmic scale. In addition, since the introduction of TyG index, its use has been increasing in biomedical literature. However, the TyG index formula has been calculated in two different ways. In some literature, the TyG index is calculated according to the following: ln(triglycerides (mg/dL) × fasting blood glucose (mg/dL)/2). Other studies have calculated the TyG index according to the following: ln(triglycerides (mg/dL) × fasting blood glucose (mg/dL))/2. The position of the division sign in the formula was inside the outer brackets when the formula was first introduced [39].

### 2.3. Frequency of Breakfast Consumption

Breakfast is defined as the first eating occasion of the day, occurring within 2 h of waking and before 10:00 a.m. [40,41]. The questionnaire about the daily meal frequency in KNHANES is divided into breakfast, lunch, and dinner. The questionnaire asks, “How many breakfasts did you have per week in the last year?” There are four responses to the question: 5–7 times per week, 3–4 times per week, 1–2 times per week, and rarely (0 times per week). Based on responses to this survey, we reclassified groups of independent variables to 5–7 times per week, 1–4 times per week, and 0 times per week. The group having breakfast 5–7 times per week was set as the reference.

### 2.4. Covariates

Covariates were socioeconomic status, health-related, and nutritional factors. The following covariates were included in the fully adjusted models because they are known to be associated with diabetes risk and could confound the relationship with diabetes risk and breakfast consumption: age (<30, 30–39, 40–49, 50–59, 60–69, and ≥70), marital status (married, single, separated, or divorced), educational attainment (high school or under, university or above), household income group (low, medium-low, medium-high, and high), region (metropolitan and rural), and occupation (white-collar, pink-collar, blue-collar, and unemployed). Household income groups were divided into quartiles using the monthly average household income. The monthly household income was calculated by dividing household income by the square root of the number of household members, which is the standard method recommended by the Organization for Economic Cooperation and Development. Occupations were categorized according to the Korean version of the Standard Classification of Occupations based on the International Standard Classification of Occupations by the International Labor Organization. We restructured the classification into four categories: white (office work), pink (sales and service), blue (agriculture, forestry, fishery, and armed forces occupation), and unemployed.

Health-related covariates included body mass index (normal + underweight (<23.0), overweight (23.0–24.9), or obese (≥25.0)), waist circumference (normal circumference or abdominal obesity), subjective health status (good, normal, or bad), smoking status (non-smoker, ex-smoker, or current smoker), frequency of alcohol consumption (never, occasionally (2–4 times a month or less), or frequently (2–3 times a week or more)), and physical activity (active or inactive). Waist circumference was determined using the Korean abdominal obesity criteria, which is 90 cm for men and 85 cm for women. Physical activity was assessed by subject reporting of moderate-intensity activity for more than 150 min, a high-intensity activity for more than 75 min, or a combination of both moderate- and high-intensity activity (1 min for high intensity and 2 min for moderate intensity) per week. Nutritional covariates included macronutrient intake (carbohydrate, protein, fat) and calorie intake per day. Macronutrient intake and calorie intake were treated as continuous variables.

### 2.5. Statistical Analysis

To perform a multistage stratified probability sampling of the KNHANES, sampling weights were applied in all data analyses. Descriptive analysis was used to examine the distribution of the general characteristics of the study population. We calculated the frequency and percentages for each variable and used chi-square tests to compare them. The statistical significance level was defined as a *p*-value < 0.05. To identify the association between breakfast frequency and insulin resistance, a multiple logistic regression analysis was performed after adjusting for socio-demographic and health-related covariates. Insulin resistance was divided into the following groups according to the median TyG index (8.5): low insulin resistance group (<8.5) and high insulin resistance group (≥8.5) [42]. In addition, to estimate valid cut-off values, the TyG index was further analyzed using the receiver operating characteristic (ROC) curve for fasting glucose disorder, with an effective cut-off value of 8.5230. Therefore, it was judged that there was no difficulty in setting the standard of the high insulin resistance group to 8.5. Odds ratios (ORs) and 95% confidence intervals (CIs) were calculated to compare those who had breakfast 5–7 times per week (referent) with those who had breakfast 1–4 times per week and 0 times per week. We performed a subgroup analysis to examine whether the groups differed according to socioeconomic status, health-related aspects, and nutritional characteristics. These covariates were selected based on the existing literature [43,44,45]. In addition, multicollinearity in this statistical model was tested using the variance inflation factors, which were all below the threshold of 10 that is generally accepted to indicate collinearity. All statistical analyses were performed using SAS 9.4 software (SAS Institute, Cary, NC, USA).

## 3. Results

The general characteristics of the study subjects are shown in Table 1. The total number of participants was 12,856, of which 6584 were considered to have low insulin resistance and 6272 were considered to have increased insulin resistance. In both groups, the frequency of breakfast consumption was 5–7 times per week for most of the participants. The differences in all other variables between insulin resistance groups were statistically significant, except for fat intake and survey year.

Table 2 shows the logistic regression analysis results adjusted after controlling for socioeconomic status, health-related aspects, and nutritional covariates. The group without breakfast had the greatest risk for increased insulin resistance. Compared with the reference group (breakfast 5–7 times per week), the odds ratios (95% CIs) for high insulin resistance were as follows: OR = 1.42 (95% CI 1.24–1.64) for breakfast 0 times per week; OR = 1.17 (95% CI 1.03–1.32) for 1–4 times per week. Men had a higher risk of insulin resistance than women (OR = 1.95 (95% CI 1.72–2.22) for men; women were set as the reference). Regarding BMI and waist circumference, obese individuals had a higher risk of high insulin resistance than the normal, non-obese individuals (OR = 2.51 (95% CI 2.17–2.91) for BMI; OR = 1.83 (95% CI 1.59–2.11) for waist circumference). Participants with poor or average subjective health had higher odds of insulin resistance than people with good subjective health (OR = 1.24 (95% CI 1.07–1.43) for bad subjective health status; OR = 1.28 (95% CI 1.16–1.42) for normal subjective health status). Smokers had higher odds of insulin resistance than non-smokers (OR=1.68 (95% CI 1.45–1.96)). People without physical activity also had higher odds of insulin resistance than active group (OR = 1.19 (95% CI 1.07–1.31)). The *p*-values for these were all significant.

Table 3 shows the results of the subgroup analyses regarding the impact of lunch and dinner consumption, sex, age, BMI, waist circumference, and physical activity on increased insulin resistance according to the number of breakfasts consumed per week. Lunch or dinner consumption and age did not have a clear pattern associated with increased insulin resistance. Both men and women who reported “never eating breakfast” were more likely to be susceptible to a higher risk of insulin resistance compared to the breakfast 5–7 times per week reference group (men: OR = 1.47, 95% CI = 1.18–1.84; women: OR = 1.37, 95% CI = 1.14–1.64), with men having a higher likelihood of being in the high-risk group compared to women. The odds ratio of the overweight or obese group was significantly higher than those of the other groups. Obese respondents tended to have higher ORs for increased insulin resistance when the frequency of breakfast consumption was ≤4 times per week compared to individuals who were underweight or had normal BMI. Particularly, individuals who did not eat breakfast at all were significantly more likely to be at higher risk (overweight: OR = 1.45, 95% CI = 1.08–1.94; obese: OR = 1.70, 95% CI = 1.29–2.24). In addition, individuals who had abdominal obesity showed higher ORs for high insulin resistance when they did not have breakfast at all each week compared with the normal group (OR = 1.50, 95% CI = 1.14–1.97). People without physical activity were more likely to have increased insulin resistance when skipping breakfast (OR = 1.52, 95% CI = 1.26–1.84) or having breakfast under 4 times per week (OR = 1.22, 95% CI = 1.04–1.43).

## 4. Discussion

It is widely believed that skipping breakfast has a negative effect on an individual’s general health [19]. The purpose of this study was to examine the association between weekly breakfast frequency and insulin resistance in the Korean population using the TyG index from the representative KNHANES data [46]. We also conducted subgroup analysis using weekly lunch and dinner frequencies, age, sex, BMI, waist circumference, and physical activity, which are factors related to insulin resistance. This is one of the few studies that has examined the relationship between breakfast and insulin resistance in Koreans through the TyG index. The TyG index has been shown to be a useful surrogate marker.

We observed that decreased weekly breakfast frequency was associated with a higher risk of insulin resistance in people without diabetes. Additionally, not eating breakfast was significantly associated with the highest probability of insulin resistance. These results are similar to those of earlier studies, confirming that not eating breakfast is a risk factor for T2DM [20,47,48]. As previous studies have shown that skipping breakfast is linked to obesity and insulin resistance [47,49,50], it is possible that recurrent breakfast skipping over a long period alters metabolism and results in increased storage of fat [30]. According to previous studies, the effects of skipping breakfast were not compensated by an increased food intake at lunchtime [51]; there is a significant association between skipping breakfast and body weight gain, insulin resistance, atherosclerotic cardiovascular disease, metabolic risk, and T2DM [23,30,52,53]. In addition, skipping breakfast might reduce 24-hour energy expenditure and elevate the level of blood glucose [20]. A study of healthy lean women found that skipping breakfast led to higher total plasma and LDL cholesterol concentrations and lower insulin sensitivity than in those women who ate breakfast [52]. Another study found that breakfast consumption aided in controlling appetite and blood glucose concentrations in children and adults [54], and that eating breakfast might reduce the risk of T2DM in men [34].

We found that men were almost twice as likely to have increased insulin resistance than women. In general, T2DM is more prevalent in men than in women, which is based on the differences in endogenous sex hormones [55]. Previous studies have reported that among men, breakfast skipping is linked to diabetes-related factors, such as high total cholesterol (TC) or low-density lipoprotein cholesterol, elevated blood pressure, low cortisol, and obesity [56,57,58]. However, some studies have reported a greater relevance to breakfast consumption and the risk of diabetes in women [58,59]. Our subgroup analysis revealed that the association between the frequency of breakfast consumption and insulin resistance was more pronounced amongst participants with obesity and abdominal obesity (*p*-values for interaction were <0.05).

This study is relevant in that it investigated the link between the frequency of breakfast consumption and insulin resistance in Koreans using the TyG index. Our study results support and extend the findings from previous studies by showing that having breakfast more than once a week can help prevent diabetes by lowering insulin resistance compared to not having breakfast at all. Therefore, we suggest that controlling insulin resistance by having breakfast might help to prevent T2DM to some extent.

The present study is important for public health because it considers the preventive aspects of T2DM, which has a high disease burden. Moreover, it clarifies the association between breakfast frequency and insulin resistance; eating breakfast is a lifestyle habit that can be easily modified for the prevention of T2DM. Our research has several strengths. First, the data analyzed were from a nationwide survey based on a random cluster sampling, which allowed our results to be reflective of the general health status in Korea. Second, this study used the TyG index, which has high predictive power, as a tool for evaluating insulin resistance [13]. According to a community-based prospective cohort study, the TyG index was a better predictor for T2DM compared with visceral adiposity index (VAI), lipid accumulation product (LAP), and HOMA-IR [60] approaches.

Despite these strengths, this study has several limitations that should be considered when interpreting the results. First, as this study was a cross-sectional study, the results cannot infer a clear causal relationship between the frequency of breakfast and insulin resistance. Because breakfast is not an intervention, the relatively low risk of insulin resistance in individuals who eat breakfast more often might be related to their general healthier lifestyles. Second, despite the efforts of the surveying agency to reduce bias, the original data we analyzed might have been affected by response bias. The data used in this study were mostly based on self-reported surveys. Therefore, questions about the frequency of meals per week, socioeconomic status, and health-related behaviors can cause recall bias. In addition, we could check the frequency of meals according to the meal constitution; however, it was not possible to determine the items or calorie intake consumed for breakfast. Because nutritional data were only given for total daily intake and the corresponding nutritional content, we could only adjust the total amount of nutritional factors per day as a covariate. Therefore, future studies would need to confirm the association between breakfast items and insulin resistance. Third, due to the nature of the data, we could not consider the frequency of breakfast divided into weekdays versus weekends in this study.

## 5. Conclusions

Our study demonstrated that breakfast skipping is negatively associated with insulin resistance, as indicated by TyG index in the Korean population. In addition, male sex, high BMI, abdominal obesity, and low physical activity tended to increase the risk of high insulin resistance. These findings indicate that regular breakfast consumption may effectively reduce the development of insulin resistance in the Korean population. Therefore, promoting the benefits of breakfast could be a simple and effective public health message to prevent diabetes.

## Figures and Tables

**Table 1:** General characteristics of the study population.

Variables	Insulin Resistance (TyG Index)						
	Total		Low (<8.5)		High (≥8.5)		*p*-Value ^4^
	N	%	N	%	N	%	
**Breakfast per week**							<0.0001
0 times	1761	13.7	919	52.2	842	47.8	
1–4 times	2960	23.0	1626	54.9	1334	45.1	
5–7 times	8135	63.3	4039	49.6	4096	50.4	
**Sex**							<0.0001
Male	5278	41.1	2029	38.4	3249	61.6	
Female	7578	58.9	4555	60.1	3023	39.9	
**Age**							<0.0001
<30	1913	14.9	1363	71.2	550	28.8	
30–39	2262	17.6	1309	57.9	953	42.1	
40–49	2532	19.7	1268	50.1	1264	49.9	
50–59	2352	18.3	1063	45.2	1289	54.8	
60–69	2045	15.9	831	40.6	1214	59.4	
≥70	1752	13.6	750	42.8	1002	57.2	
**Marital status**							<0.0001
Single, separated, divorced	4041	31.4	2270	56.2	1771	43.8	
Married	8815	68.6	4314	48.9	4501	51.1	
**Educational level**							<0.0001
College or over	6155	47.9	3452	56.1	2703	43.9	
Highschool or less	6701	52.1	3132	46.7	3569	53.3	
**Household income**							<0.0001
Low	2081	16.2	954	45.8	1127	54.2	
Lower middle	3111	24.2	1550	49.8	1561	50.2	
Upper middle	3666	28.5	1923	52.5	1743	47.5	
High	3998	31.1	2157	54.0	1841	46.0	
**Region**							0.0277
Rural	7131	55.5	3590	50.3	3541	49.7	
Metropolitan	5725	44.5	2994	52.3	2731	47.7	
**Occupational categories ^1^**							<0.0001
White-collar	3391	26.4	1829	53.9	1562	46.1	
Pink-collar	1657	12.9	870	52.5	787	47.5	
Blue-collar	2816	21.9	1281	45.5	1535	54.5	
Unemployed or other	4992	38.8	2604	52.2	2388	47.8	
**BMI ^2^**							<0.0001
Obese (≥25.0)	4091	31.8	1345	32.9	2746	67.1	
Overweight (23.0–24.9)	2911	22.6	1301	44.7	1610	55.3	
Underweight or Normal (<23.0)	5854	45.5	3938	67.3	1916	32.7	
**Waist circumference ^3^**							<0.0001
Abdominal obesity	4524	35.2	1353	29.9	3171	70.1	
Normal	8330	64.8	5231	62.8	3099	37.2	
**Subjective health status**							<0.0001
Bad	2148	16.7	997	46.4	1151	53.6	
Normal	6711	52.2	3306	49.3	3405	50.7	
Good	3997	31.1	2281	57.1	1716	42.9	
**Smoking status**							<0.0001
Current smoker	2089	16.2	701	33.6	1388	66.4	
Ex-smoker	2576	20.0	1087	42.2	1489	57.8	
Non-smoker	8191	63.7	4796	58.6	3395	41.4	
**Drinking status**							<0.0001
Frequently	2755	21.4	1127	40.9	1628	59.1	
Occasionally	6714	52.2	3715	55.3	2999	44.7	
Never	3387	26.3	1742	51.4	1645	48.6	
**Physical activity**							<0.0001
Inactive	7044	54.8	3409	48.4	3635	51.6	
Active	5812	45.2	3175	54.6	2637	45.4	
**Lunch per week**							<0.0001
0 times	210	1.6	98	46.7	112	53.3	
1–4 times	1103	8.6	574	52.0	529	48.0	
5–7 times	11,543	89.8	5912	51.2	5631	48.8	
**Dinner per week**							<0.0001
0 times	59	0.5	31	52.5	28	47.5	
1–4 times	1262	9.8	733	58.1	529	41.9	
5–7 times	11,535	89.7	5820	50.5	5715	49.5	
Calorie intake (kcal/day) *	1937.5	± 879.9	1879.6	± 848.4	1998.4	± 907.9	<0.0001
Carbohydrate intake (g/day) *	294.4	± 123.9	286.5	± 121.4	302.7	± 125.9	<0.0001
Protein intake (g/day) *	70.6	± 40.5	69.5	± 39.5	71.7	± 41.6	0.003
Fat intake (g/day) *	45.1	± 35.0	45.7	± 35.1	44.5	± 35.0	0.056
**Year**							0.3168
2016	4323	33.6	2254	52.1	2069	47.9	
2017	4201	32.7	2126	50.6	2075	49.4	
2018	4332	33.7	2204	50.9	2128	49.1	
**Total**	12,856	100.0	6584	51.2	6272	48.8	

^1^ Three groups (white, pink, blue) based on the International Standard Classification Occupations codes. Unemployed group includes housewives. TyG: triglyceride-glucose; ^2^ BMI: body mass index; obesity status defined by BMI based on the 2018 Clinical Practice Guidelines for Overweight and Obesity in Korea. ^3^ Waist circumference was determined using the Korean abdominal obesity criteria, which is 90 cm for men and 85 cm for women. ^4^ Significant *p*-value is <0.05. * Values are presented as mean ± standard deviation. Note: *p*-values were obtained from the chi-square test and t-test.

**Table 2:** Odds ratio for insulin resistance.

Variables	Insulin Resistance		
	OR ^1^	95% CI	*p*-Value *^7^*
**Breakfast per week**			
0 times	1.42	(1.24–1.64)	<0.0001
1–4 times	1.17	(1.03–1.32)	0.0153
5–7 times	1.00		
**Sex**			
Male	1.95	(1.72–2.22)	<0.0001
Female	1.00		
**Age**			
<30	1.00		
30–39	1.91	(1.59–2.29)	<0.0001
40–49	2.90	(2.41–3.48)	<0.0001
50–59	3.54	(2.90–4.32)	<0.0001
60–69	3.82	(3.06–4.77)	<0.0001
≥70	3.20	(2.52–4.07)	<0.0001
**Marital status**			
Single, separated, divorced	1.17	(1.03–1.32)	0.0143
Married	1.00		
**Educational level**			
College or over	1.02	(0.91–1.14)	0.7084
Highschool or less	1.00		
**Household income**			
Low	1.00	(0.85–1.19)	0.8698
Lower middle	1.02	(0.86–1.22)	0.8620
Upper middle	1.02	(0.85–1.21)	0.8982
High	1.00		
**Region**			
Rural	1.00	(0.90–1.10)	0.9723
Metropolitan	1.00		
**Occupational categories ^2^**			
White-collar	0.91	(0.81–1.03)	0.1352
Pink-collar	0.87	(0.74–1.01)	0.0589
Blue-collar	0.66	(0.58–0.76)	<0.0001
Unemployed or other	1.00		
**BMI ^3^**			
Obese (≥25.0)	2.51	(2.17–2.91)	<0.0001
Overweight (23.0–24.9)	1.85	(1.63–2.09)	<0.0001
Underweight or Normal (<23.0)	1.00		
**Waist circumference ^4^**			
Abdominal obesity	1.83	(1.59–2.11)	<0.0001
Normal	1.00		
**Subjective health status**			
Bad	1.24	(1.07–1.43)	0.0048
Normal	1.28	(1.16–1.42)	<0.0001
Good	1.00		
**Smoking status**			
Current smoker	1.68	(1.45–1.96)	<0.0001
Ex-smoker	1.05	(0.91–1.20)	0.5072
Non-smoker	1.00		
**Drinking status**			
Frequently	1.13	(0.97–1.31)	0.1196
Occasionally	0.92	(0.82–1.04)	0.1767
Never	1.00		
**Physical activity**			
Inactive	1.19	(1.07–1.31)	0.0011
Active	1.00		
**Lunch per week**			
0 times	0.92	(0.65–1.31)	0.6493
1–4 times	1.07	(0.90–1.28)	0.4239
5–7 times	1.00		
**Dinner per week**			
0 times	1.11	(0.58–2.11)	0.7609
1–4 times	0.90	(0.77–1.05)	0.1751
5–7 times	1.00		
**Carbohydrate intake (g/day) ^5^**	0.999	(0.998–1.000)	0.0083
**Protein intake (g/day) ^5^**	0.996	(0.994–0.998)	0.0009
**Fat intake (g/day) ^5^**	0.996	(0.993–0.998)	0.0015
**Calorie intake (kcal/day) ^6^**	1.000	(1.000–1.001)	<0.0001

^1^ OR: adjusted odds ratio. Socioeconomic status, health-related aspects, and nutritional covariates were adjusted. ^2^ Three groups (white, pink, blue) based on the International Standard Classification Occupations codes. Unemployed group includes housewives. ^3^ BMI: body mass index; obesity status defined by BMI based on the 2018 Clinical Practice Guidelines for Overweight and Obesity in Korea. ^4^ Waist circumferences was determined using the Korean abdominal obesity criteria, which is 90 cm for men and 85 cm for women. ^5^ Per 1 (g) increase. ^6^ Per 1 (kcal) increase. ^7^ Significant *p*-value is <0.05.

**Table 3:** Results of subgroup analysis for the association between weekly breakfast frequency and insulin resistance according to different factors.

Variables		Insulin Resistance					
	5–7	0			1–4		
	OR ^1^	OR ^1^	95% CI	*p*-Value ^5^	OR ^1^	95% CI	*p*-Value ^5^
**Sex**							
Male	1.00	1.47	(1.18–1.84)	0.0007	1.18	(0.96–1.44)	0.1214
Female	1.00	1.37	(1.14–1.64)	0.001	1.15	(0.98–1.35)	0.0805
**Age**							
<30	1.00	1.61	(1.16–2.25)	0.0048	1.16	(0.84–1.35)	0.3650
30–39	1.00	1.34	(0.99–1.80)	0.0582	1.18	(0.91–1.53)	0.2163
40–49	1.00	1.75	(1.28–2.38)	0.0004	1.42	(1.10–1.83)	0.0065
50–59	1.00	1.25	(0.88–1.76)	0.2096	0.84	(0.63–1.13)	0.2450
60–69	1.00	0.91	(0.54–1.56)	0.7385	1.20	(0.79–1.83)	0.4025
≥70	1.00	1.15	(0.50–2.63)	0.7453	1.01	(0.58–1.76)	0.9732
**BMI ^3^**							
Obese	1.00	1.70	(1.29–2.24)	0.0002	1.16	(0.93–1.46)	0.1926
Overweight	1.00	1.45	(1.08–1.94)	0.0137	0.90	(0.69–1.17)	0.4195
Underweight or Normal	1.00	1.31	(1.07–1.61)	0.0092	1.36	(1.14–1.64)	0.0009
**Waist circumference ^4^**							
Abdominal obesity	1.00	1.50	(1.14–1.97)	0.0040	1.15	(0.92–1.43)	0.2332
Normal	1.00	1.42	(1.20–1.68)	0.0001	1.19	(1.02–1.38)	0.0234
**Physical activity**							
Inactive	1.00	1.52	(1.26–1.84)	<0.0001	1.22	(1.04–1.43)	0.0172
Active	1.00	1.34	(1.08–1.66)	0.0073	1.12	(0.93–1.34)	0.2475
**Lunch per week**							
0 times	1.00	7.31	(1.50–35.7)	0.0141	1.59	(0.39–6.46)	0.5155
1–4 times	1.00	1.28	(0.76–2.13)	0.3508	1.13	(0.78–1.64)	0.5093
5–7 times	1.00	1.41	(1.21–1.63)	<0.0001	1.16	(1.02–1.32)	0.0296
**Dinner per week**							
0 times	1.00	–^2^	–	–	–^2^	–	–
1–4 times	1.00	1.24	(0.81–1.89)	0.3190	0.93	(0.66–1.33)	0.6966
5–7 times	1.00	1.45	(1.25–1.68)	<0.0001	1.20	(1.05–1.36)	0.0087

^1^ OR: adjusted odds ratio. Socioeconomic status, health-related aspects, and nutritional covariates were adjusted. ^2^ Not calculated due to the small number of subjects. ^3^ BMI: body mass index; obesity status defined by BMI based on the 2018 Clinical Practice Guidelines for Overweight and Obesity in Korea. ^4^ Waist circumference was determined using the Korean abdominal obesity criteria, which is 90 cm for men and 85 cm for women. ^5^ Significant *p*-value is <0.05.
